# Ferruginous Pill of Mercury

**Published:** 1845-07

**Authors:** John McLean

**Affiliations:** Jackson


### ILLINOIS

### MEDICAL & SURGICAL JOURNAL.

VOL. II. JULY, 1845. NO. 4.

### FERRUGINOUS PILL OF MERCURY.

. In a number of the London Lancet, for 1843, I observed a formula for preparing mercurial pills, which is as follows:

" ℞ Ferri. sesquioxydi ℥i.

Hydrargyri ℥ii.

Confect. Rosæ Gallicæ ℥iii.

Contere donec Globuli non Omplias conspicantur."

Prepared in the above manner, the mass is not of sufficient consistence to form into pills. The following is the method which I have adopted:

| | |
|---|---|
| Mercury, | 1 oz. |
| Confection of Roses, | 1½ oz. |
| Sesquioxide of Iron, | ½ oz. |
| Liquorice Root, in powder, | ½ oz. |

Mix the iron and confection of roses, then add the mercury, and rub till the globules disappear; lastly, add the liquorice, and thoroughly incorporate it into the mass.

A much less quantity of iron, than is here given, will answer for the speedy and effectual reduction of the mercury. I have formed the mass, as speedily and perfectly with one-fourth, as with the amount given in the formula. If in any instance, it is desirable that an article containing less, should be used, it can easily be prepared accordingly. There are a few cases, where the Iron might be objectionable, but on the other hand, there are many where it would act beneficially as a remedial agent, in this connexion with mercury.

The mercurial pill is in such common use, and of so much acknowledged utility in many cases of disease, that any improvement in its preparation, becomes an object of interest to the profession. Owing to the difficulty of preparing it, after the formula of the Pharmacopœia, many practitioners prefer purchasing it of the apothecary, and an inferior article not to be relied on, is often procured, and thus the looked for results of their prescriptions, are not realized. It is all important, that Physicians should be acquainted with the quality of their remedies, in order to treat successfully, disease. By the addition of the Iron, the great labor of forming the blue pill is done away with, so that every physician can prepare his own. What required hours of labor, by the method usually adopted, may be accomplished, by this new form, in ten minutes. I have, in from 5 to 10 minutes, so thoroughly reduced the mercury, that no globules could be discovered by the aid of a glass, magnifying from 10 to 15 times. Should the physician still prefer to purchase of the apothecary, (if he would prepare after the foregoing manner,) a more uniform article, and of a superior efficacy would be received.

Sulphuric acid is sometimes added to the conserve of roses to improve its color, which, if present, when the mercurial pill is prepared according to the usual method, forms a subsulphate of mercury—a compound possessing very energetic properties; but when formed with the oxide of Iron, instead of this, a harmless ferruginous salt is produced, and the uniformity of the mercurial compound is not disturbed. Mr. Abernethy, in speaking of the uncertainty of the blue pill in its operation, seems to think, that this may depend on the sulph. acid, which frequently is found to exist in the conserve of Roses. Dr. Paris observes: "It is not improbable, that in making the conserve for sale, some of this acid may be added to brighten the color; and if so, the mercurial pill, which is made from it, may contain in varying portions, some of that highly deleterious compound, the subsulphate of mercury."

Again, when prepared after the usual manner, the strength is liable to vary, on account of the difficulty of reducing the mercury, for which reason it is many times left in an imperfectly finished condition; but by the method now offered, this difficulty is entirely obviated. There have been different theories with regard to the manner in which the oxide of Iron assists in the reduction of the mercury; but at present we shall not enter upon the discussion of this point, but content ourselves with the fact that it assists very materially in the formation of the mercurial pill. Lastly, I believe this preparation to be superior to the usual one, in all cases where Iron is not contra-indicated. I have now used it for nearly two years, and have found it much more satisfactory than the other. According to my experience, it is more energetic as a cathartic, and acts with more promptitude and certainty upon the secretions. In cases of Anemia and protracted intermittents, when mercurials are indicated, this preparation is peculiarly applicable, in consequence of the Iron it contains.

The advantages which this possesses over the common blue pill, may be summed up as follows: 1st. It is much more easily prepared. 2nd. Its strength is more uniform. 3d. It is more active and uniform in its operation.

Jackson, June, 1845. JOHN McLEAN.

