# Erratum

**Published:** 1845-08

**Authors:** 


*Erratum.*—On page 49 of our last number, (July,) for "*omplias conspicantur*" read "*amplias conspiciantur.*"

